# Effects of cervical stabilization with visual feedback on craniovertebral angle and proprioception for the subjects with forward head posture

**DOI:** 10.1097/MD.0000000000036845

**Published:** 2024-01-12

**Authors:** Bon Wook Goo, Jin Hee Oh, Ju Sang Kim, Mi Young Lee

**Affiliations:** a Rehabilitation Center, Yeungnam University Medical Center, Daegu, Republic of Korea; b Department of Medical Science, Graduate School, Daegu Haany University, Gyeongsan, Republic of Korea; c Department of Physical Therapy, Yeungnam University of College, Daegu, Republic of Korea; d Department of Physical Therapy, College of Biomedical Science, Daegu Haany University, Gyeongsan, Republic of Korea.

**Keywords:** craniovertebral angle, forward head posture, proprioception, stabilization exercise, visual feedback

## Abstract

**Background:**

This study aimed to identify the effect of cervical stabilization exercise with visual feedback on the craniovertebral angle and proprioception in subjects with forward head posture.

**Methods:**

Thirty healthy adults were recruited in the study. Participants were randomly assigned to the stabilization exercise with visual feedback (SE-VF) group (N = 15) or stabilization exercise group (N = 15). The SE-VF group performed cervical stabilization exercise while sitting on a chair without a backrest and checking their side profile in real-time via a monitor 3-m away. The stabilization exercise group performed the same cervical stabilization exercise as the SE-VF group but without visual feedback. Craniovertebral angle (CVA) was measured to quantify forward head posture, and the proprioception of the subjects was evaluated.

**Results:**

There was a significant interaction between group and time in CVA and proprioception (*P* < .05). Additionally, there was no significant difference pre-intervention between the groups (*P* > .05); however, there was a significant difference post-intervention (*P* < .05) in CVA and proprioception.

**Conclusion:**

The findings of this study showed that the cervical stabilization exercise with visual feedback was effective for the proprioception of subjects. Moreover, the results suggest that visual feedback is effective in cervical stabilization exercise.

## 1. Introduction

The contemporary trend of increased computer usage, coupled with prolonged desk-based working, continued use of abnormal postures, and lack of exercise, has increased muscular stiffness in the neck and shoulders,^[1]^ putting significant stress on the posterior skeletal structures.^[2]^ Consequently, such abnormal postures lead to cervical hyperextension, anterior positioning of the head, and the development of forward head posture (FHP).^[3]^

FHP alignment is commonly observed as the most prevalent posture deviation in the sagittal plane, characterized by the head being excessively forward as compared to the shoulders.^[4]^ As the center of gravity of the head is positioned anteriorly to the vertical axis, it leads to an increased load on the posterior neck and results in excessive extension of the upper cervical spine (C1–C3) and flexion of the lower cervical spine (C4–C7).^[5,6]^ FHP imposes approximately 3.6 times more pressure on the neck than proper posture, and the prolonged burden of supporting the increased head weight can potentially cause postural dislocation, neck pain, fatigue, and chronic musculoskeletal disorders.^[5]^

Cervical muscles are densely populated with muscle spindles, which are sensory receptors that detect changes in muscle length, requiring a high proprioceptive capacity.^[7]^ In particular, the suboccipital muscles harbor a significant number of muscle spindles,^[8]^ with over 200 spindles per gram of muscle tissue.^[9]^ Hence, alterations in the length of these neck muscles can potentially lead to abnormalities in the inherent proprioceptive function of the cervical spine.^[10,11]^

The proprioceptive function is crucial for the proper functioning of joints during exercise and everyday activities. It provides dynamic stability to the joints; specifically, the proprioceptive function in the neck plays a vital role in providing information regarding neck posture and movement to the central nervous system.^[12]^ It has been reported that proprioceptive function regulates posture-related balance and that patients with neck pain exhibit increased positional errors for neck joints and reduced balance.^[13]^ Therefore, the restoration of proprioceptive function is probably crucial for the proper regulation and maintenance of normal neck posture in FHP.

To address underlying soft tissue imbalances in individuals with FHP, it is recommended to employ therapeutic approaches that strengthen weakened postural muscles and stretch shortened muscles to improve postural alignment.^[5]^ Specifically, exercises to strengthen scapular retractors and deep cervical flexors,^[14]^ manual therapy and stabilization exercises,^[15]^ isometric exercises and stretching techniques,^[16]^ pressure biofeedback,^[17]^ and McKenzie exercises^[18]^ are used to prevent and treat various symptoms. Cervical stabilization exercises are interventions that strengthen the deep cervical flexors and enhance coordination between superficial and deep cervical muscles; they have gained popularity in recent years as an intervention for individuals with FHP.^[19]^ Furthermore, cervical stabilization exercises are used as a rehabilitation intervention for patients with chronic neck pain to strengthen their core spinal muscles and enhance endurance and coordination.^[20]^

Vision is an important body function that provides information about the body’s posture and movement, as well as the surrounding environment, and is crucial in determining the level of neuromuscular activity.^[21]^ Previous research demonstrated that movement accuracy in the proprioceptive-visual condition was significantly higher than the proprioceptive-only condition and slightly higher than the vision-only condition.^[22]^ Additionally, visual feedback (VF) may stimulate skill acquisition and regulation of the neuromuscular system through additional sensory input.^[23]^ Previous studies have reported the effectiveness of visual feedback in stimulating functional recovery and improvement in various rehabilitation area.^[24–26]^

However, there is limited previous study on the effectiveness of visual feedback in correcting of posture. Thus, this study aimed to investigate the effects of cervical stabilization exercises coupled with visual feedback training on the craniovertebral angle (CVA) and proprioception in individuals with FHP.

## 2. Materials and methods

### 2.1. Study participants

**Figure 1.**
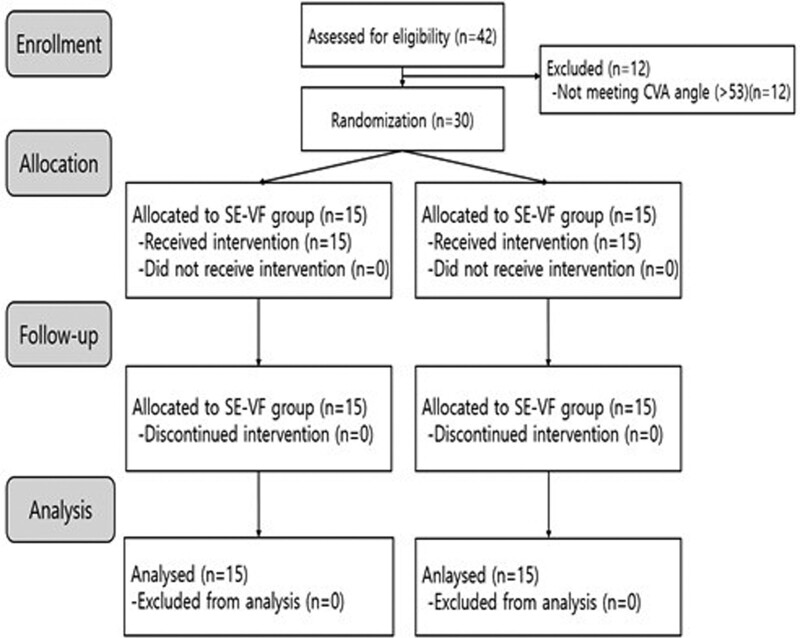
Flow chart of participants.

Thirty healthy adults with FHP who met the eligibility criteria of this study were enrolled. The inclusion criteria were: age 19 to 28 years, right-hand dominance, CVA ≤ 53º,^[27–29]^ and vertical distance between the tragus and acromion ≤ 3.5 cm. The exclusion criteria were: history of spinal or thoracic surgery, structural spinal malformation, history of surgical or neurologic disorders, and treatment for cervical trauma or pain in the past 6 months. The participants were randomly assigned with allocation concealment into the stabilization exercise-visual feedback (SE-VF) (n = 15) and SE groups (n = 15). For the randomized allocation, we generated random numbers using the Microsoft Excel program. The subjects and assessor were blinded to the subject group assignment. A flowchart of the trail design is shown in Figure 1. This study was conducted in adherence with the declaration of Helsinki and was approved by the Institutional Review Board at Daegu Haany University (DHU 2021-1-01). The participants were given an adequate explanation about the purpose and method of the study and signed a written informed consent before participating in the study.

### 2.2. Experimental method

#### 2.2.1. Craniovertebral angle.

To measure CVA, a photograph of the side of the head was captured from 3 m away from the participants. The angle was measured from the photographs using Photoshop (Adobe Inc., San Jose, CA). The CVA is a commonly used method for assessing FHP, and it refers to the angle between the horizontal line at the level of C7 and the line connecting C7 to the midpoint of the tragus. Although there is no clear cutoff line threshold that accurately indicates FHP for CVA, in our study, we defined FHP as CVA of ≤53º based on previous studies.^[27–30]^

#### 2.2.2. Proprioception.

In this study, we measured the position-reposition error using a portable digital inclinometer (Acumar dual digital inclinometer, Lafayetter Instrument, IN) to evaluate the participants’ proprioception. Position-reposition error is a common test used to assess proprioception. During the test, a reference position is established, and the participant is instructed to move their joint to the maximum range of motion (ROM) and then to reproduce the reference position. The difference between the reference position and the position reproduced by the participant is used to assess proprioceptive function.^[31]^ The portable digital inclinometer consists of 2 main components: a companion unit that allows reference point setting and a main unit that displays the measured values on an LCD screen. It is primarily used to measure the ROM in limb joint and is reported to have high reliability in assessing spinal flexion ROM and posture evaluation.^[32]^ In the measurement process, one side of the digital inclinometer (companion unit) was fixed to the participant’s C7 vertebra, while the other side (main unit) was placed near the top of the head to read the measured values. The joint position-reposition error test was performed in a self-balance posture. The self-balance posture is a posture in which the participant’s head is positioned in the most comfortable position after performing maximal cervical flexion and extension.^[33]^

### 2.3. Intervention

**Figure 2.**
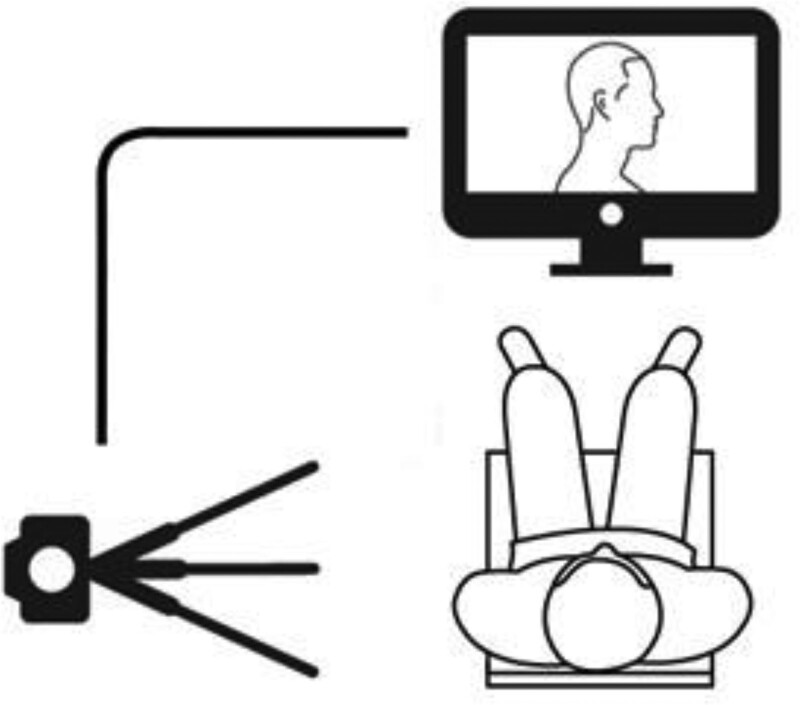
Method of cervical stabilization exercise with visual feedback. The participant sits on a backless chair to have their side view photographed. They performed cervical stabilization exercise while monitoring their posture in real-time from a monitor placed 3 m in front of them.

The 30-minute exercise program for the 2 groups consisted of a warm-up, cervical SE, and cool-down. The warm-up involved 5 exercises for 5 minutes: rotating wrist and ankle, posterior shoulder stretching, cervical flexor stretching, and wrist stretching. The SE-VF group had their side images taken while sitting on a backless chair, and they continued to perform the SE on the chair while monitoring their posture in real-time through a monitor placed 3 m in front of them. The SE group performed the same SE regimen without the VF (Fig. 2).

The SE program consisted of 4 weeks of exercise. In week 1, each rep consisted of chin tuck for 10 seconds followed by a 10-second rest while sitting on a backless chair, and 3 sets of 10 reps were performed. In week 2, a shoulder flexion exercise using a theraband was added during a chin tuck, and 3 sets of 10 reps were performed. From week 3, the same exercise as week 2 was performed on an unstable surface, specifically, a gym ball instead of a backless chair. The SE program lasted for 20 minutes including rests. Chest stretching exercises consisted of 3 movements (upper, middle, and lower stretches). Participants leaned against a wall with arms spread apart and held the stretching position for 20 seconds, and 3 sets were performed. The cool-down exercises were performed at a low intensity, focusing on regulating breathing. The stretching exercises from the warm-up session were performed for 5 minutes.^[34]^

Both groups performed 4 30-minute sessions a week, with a total of 16 sessions over 4 weeks. CVA and proprioception were measured before and after the intervention.

### 2.4. Sample size

The sample size was calculated using G*power 3.1.9.7 (Franz Faul, Kiel, Germany) based on previously published data with CVA as the primary outcome.^[35]^ Therefore, we calculated that the sample size of 14 participants per group would be required to provide 80% power at an alpha level of 0.05. Considering the dropout rate of 5%, a total of 30 individuals (15 in each group) were recruited.

### 2.5. Statistical analysis

SPSS version 25 for Windows (IBM Corporation, New York) was used for analyzing the results. We used the Shapiro–Wilk test to test for normality. Statistical data were expressed as means and standard deviations. To investigate the interaction of group and time, a two-way repeated-measures ANOVA was conducted. In addition, an independent *t*-test was used to determine the pre- and post-intervention difference between the groups. A significance level of 0.05 was used for the statistical tests.

## 3. Results

### 3.1. Participant’s general characteristics

Thirty individuals with FHP were enrolled in the study, randomized to the SE-VF and SE groups (n = 15 each). There were no significant differences in sex, age, height, and weight between the 2 groups (*P* > .05). The participants’ general characteristics are visible in Table 1.

**Table 1:** General characteristics and homogeneity of the subjects.

	SE-VF group	SE group	t	*P*
	Mean ± SD	Mean ± SD		
Sex (M/F)	9/6	9/6		1.000
Age (years)	23.13 ± 1.88	23.47 ± 1.30	0.401	.532
Height (cm)	169.07 ± 6.72	170.73 ± 8.42	0.299	.589
Weight (kg)	66.27 ± 11.07	66.67 ± 12.32	0.088	.769

SE group = stabilization exercise group, SE-VF group = stabilization exercise with visual feedback group.

### 3.2. Craniovertebral angle

There was a significant interaction between group and time (F(1,28) = 9.40, *P* = .005). There was a simple effect on CVA in both the SE-VF group (F(1,14) = 74.96, *P* = .000) and SE group (F(1,14) = 16.50, *P* = .001). Considering pre- and post-intervention differences between the groups, there was no significant difference pre-intervention between the groups (*P* > .05); however there was a significant difference post-intervention (*P* < .05) (Table 2).

**Table 2:** Comparison of CVA and proprioception between pre and post intervention.

			SE-VF group	SE group	*F*
			Mean ± SD	Mean ± SD	
CVA (°)		Pre	45.89 ± 3.87	45.57 ± 4.83	9.397*
		Post	52.21 ± 4.61†	48.66 ± 4.44†^,^‡	
Proprioception (°)	Flexion	Pre	0.40 ± 0.16	0.44 ± 0.16	9.595*
		Post	0.20 ± 0.07†	0.41 ± 0.14‡	
	Extension	Pre	0.48 ± 0.18	0.50 ± 0.18	5.894*
		Post	0.23 ± 0.09†	0.40 ± 0.16†^,^‡	

* Interaction between time and group (*P* < .05).

† Simple effect of the intervention on the interaction between time and group (*P* < .05).

‡ Significant difference between groups by the independent *t*-test (*P* < .05).

### 3.3. Proprioception

A significant interaction between group and time was seen in all conditions (flexion, F(1,28) = 9.595, *P* = .004, extension, F(1,28) = 5.894, *P* = .022). The SE-VF group demonstrated a simple effect of the intervention in all conditions (flexion, F(1,14) = 18.593, *P* = .001, extension, F(1,14) = 20.6447, *P* = .000). The SE group did not show the simple effect of the intervention during flexion, (F(1,14) = 0.798, *P* = .387), but did during extension, (F(1,14) = 7.614, *P* = .015). In the pre- and post-intervention differences between the groups, there was no significant difference pre-intervention between the groups (*P* > .05), but there was a significant difference post-intervention (*P* < .05) (Table 2).

## 4. Discussion

Recently, the prolonged use of electronic devices, such as computers and smartphones, has increased the use of abnormal postures, consequently leading to an increased number of people with spinal misalignment and joint pain.^[36]^ FHP is the most common type of abnormal posture and is considered a primary cause of musculoskeletal disorders.^[37]^ FHP places excessive strain on the neck and shoulder muscles. This hinders effective biomechanical functioning, leading to soft tissue weakening and increased fatigue.^[38,39]^ Additionally, maintaining FHP for extended periods creates a cycle of pain and discomfort, resulting in tissue overload and functional impairment.^[40]^

This study investigated the effects of SE coupled with VF on CVA and proprioception in individuals with FHP. The participants engaged in 30-minute exercises 4 times a week for 4 weeks. The following results were obtained. First, both the SE-VF group and SE group showed significant changes in CVA after the intervention. Second, the SE-VF group showed significant changes in joint position-reposition error for flexion and extension after the intervention, while the SE group only showed significant changes in the joint position-reposition error for extension. Third, between the 2 groups, there was a significant difference in CVA and proprioception only after the intervention; that is, the SE-VF group showed more improvement than the SE group. Thus, the results of our study suggest that VF likely contributed to improving CVA and proprioception during SE.

CVA is a reliable indicator for differentiating FHP and is one of the most commonly used means for assessing forward head posture.^[30]^ CVA is the angle between the line that connects the tragus of the ear to a horizontal line through the C7 vertebra.^[41]^ Furthermore, the head is considered to be balanced above the cervical spine when the tragus is positioned above the acromion process of the scapula. As the head functions as a lever of the body, a balanced head position above the cervical spine allows quick and prompt movement.^[42]^ In this study, we examined increased CVA after the SE with VF; this intervention is believed to have contributed to the enhanced body alignment in FHP. Diab et al (2012) reported that the CVA was significantly increased after 10 weeks of stretching and muscle strengthening exercise in individuals with FHP.^[43]^ Therefore, CVA could be enhanced through various exercises in individuals with FHP.

Proprioception plays a role in maintaining a proper body alignment by providing sensory feedback to the nervous system. Cervical muscles particularly play a crucial role in proprioception, as they contain a high density of muscle spindles.^[13]^ In particular, the suboccipital muscles have a high concentration of proprioceptive receptors per unit, indicating a need for high proprioceptive function.^[7]^ Proprioception is a sensory component of the body that encompasses various sensations, including force, weight, position, pressure, and movement.^[44]^ Improper positioning of the head and neck joints in individuals with a FHP negatively affects neck proprioceptive function.^[45]^ As a result, individuals with FHP may experience impaired proprioception due to abnormal alignment of the neck and head. In this study, the joint position-reposition error, which is the discrepancy between an initial position at balance and reproduced position after performing maximum flexion and maximum extension, was reduced after the SE. This finding suggests that cervical SE coupled with VF contributed to enhancing proprioception.

The effects of cervical SE have been reported extensively in the literature. Dusunceli et al (2009) reported that neck pain and neck disability index were significantly improved after 3 weeks of cervical SE followed by continued independent exercise.^[20]^ Cervical SE using a Swiss ball is utilized as an intervention for stroke and postural correction that can enhance spinal stability, impaired proprioception, and weakened neck muscle activities.^[46]^ Our results suggest that cervical SE also contributes to improving the CVA and proprioception.

VF training, applied in this study, utilizes various types of visual information, and it is widely and diversely used as an intervention to facilitate functional recovery in stroke patients.^[26]^ VF has been used in balance training and gait symmetry training in the rehabilitation of stroke patients, and VF was reported to be effective in the rehabilitation of stroke patients by providing diverse pieces of information about a task.^[47–49]^ Moreover, some studies have reported that external feedback, such as VF about arm position or standing posture, may help with motor recovery in stroke patients.^[50–52]^ In this study, real-time VF regarding postural alignment during cervical SE enhanced exercise accuracy, thereby boosting exercise efficacy.

This study has some limitations. First, we could not investigate the effects of clinical relevance such as pain reduction, functional improvement, and improved quality of life because only people with asymptomatic mild FHP were enrolled. Second, the study focusing on reducing the CVA angle without using X-rays resulted in not being able to determine whether it was effective for cervical spine alignment accurately. Future studies are required to address these limitations and investigate the effects of cervical SE with VF on several types of symptoms in individuals with FHP.

## 5. Conclusion

Cervical SE coupled with VF is more effective than conventional cervical SE for improving forward head posture and proprioceptive function. Our findings suggested that cervical SE with VF is a suitable exercise for subjects with FHP.

## Acknowledgments

The authors are grateful to all volunteer participants for their co-operation and participation in this study.

## Author contributions

**Conceptualization:** Bon Wook Goo, Ju Sang Kim.

**Data curation:** Jin Hee Oh.

**Formal analysis:** Bon Wook Goo, Mi Young Lee.

**Funding acquisition:** Mi Young Lee.

**Methodology:** Bon Wook Goo, Ju Sang Kim, Mi Young Lee.

**Resources:** Bon Wook Goo, Ju Sang Kim, Mi Young Lee.

**Supervision:** Mi Young Lee.

**Validation:** Mi Young Lee.

**Writing – original draft:** Bon Wook Goo, Jin Hee Oh.

**Writing – review & editing:** Ju Sang Kim, Mi Young Lee.
